# Treosulfan-Based Conditioning Regimens for Allogeneic Hematopoietic Cell Transplantation in Acute Myeloid Leukemia and Other Myeloid Malignancies

**DOI:** 10.3390/ph18111631

**Published:** 2025-10-29

**Authors:** Eleni Gavriilaki, Panagiotis Dolgyras, Ioannis Konstantinidis, Despina Mallouri, Grigorios Salvaras, Christos Demosthenous, Ioannis Batsis, Anna Vardi, Ioannis Papadopoulos, Sophia Tsokkou, Zoi Bousiou, Giorgos Karavalakis, Christos Varelas, Alkistis Panteliadou, Nikolaos Spyridis, Antonia Syrigou, Anastasia Marvaki, Maria Papathanasiou, Apostolia Papalexandri, Chrysavgi Lalayanni, Chrysanthi Vadikoliou, Anastasia Athanasiadou, Ioanna Sakellari

**Affiliations:** 1 BMT Unit, Hematology Department, G Papanicolaou Hospital, 57010 Thessaloniki, Greece; elenicelli@yahoo.gr (E.G.); dmallouri@gmail.com (D.M.); grigorios.salvaras@gmail.com (G.S.); christosde@msn.com (C.D.); iobats@yahoo.gr (I.B.); anna_vardi@yahoo.com (A.V.); johannpap123@gmail.com (I.P.); boussiou_z@hotmail.com (Z.B.); giorgos.karavalakis@gmail.com (G.K.); varelaschris@gmail.com (C.V.); kirapanteliadou@gmail.com (A.P.); spyridisnik@hotmail.com (N.S.); antonia.syrigou@gmail.com (A.S.); marvnat@hotmail.com (A.M.); ppthsmr@gmail.com (M.P.); lila.papalexandri@gmail.com (A.P.); luizana6@gmail.com (C.L.); crysvad@hotmail.com (C.V.); anastasiaathanas@yahoo.gr (A.A.); ioannamarilena@gmail.com (I.S.); 2 2nd Propaedeutic Department of Internal Medicine, Ippokratio University Hospital, Department of Medicine, Faculty of Health and Sciences, Aristotle University of Thessaloniki, 54124 Thessaloniki, Greece; 3 Department of Medicine, Faculty of Health Sciences, Aristotle University of Thessaloniki, 54124 Thessaloniki, Greece; ikonsc@auth.gr (I.K.); stsokkou@auth.gr (S.T.)

**Keywords:** allogeneic hematopoietic cell transplantation (allo-HCT), treosulfan, reduced-toxicity conditioning, acute myeloid leukemia (AML), myelodysplastic syndrome (MDS), myelofibrosis (MF), graft-versus-host disease (GvHD), treatment-related mortality (TRM)

## Abstract

**Background:** Treosulfan combined with fludarabine (FluTreo) has emerged as a reduced-toxicity alternative to conventional myeloablative conditioning in allogeneic hematopoietic cell transplantation (allo-HCT) for acute myeloid leukemia (AML) and related myeloid malignancies. **Purpose:** This study evaluates the safety, engraftment kinetics, and long-term outcomes of the FluTreo FT14 regimen in a real-world adult cohort. **Materials and Methods:** We conducted a prospective cohort study of 186 consecutive adults (18–70 years) undergoing allo-HCT between January 2015 and December 2024. Eligible diagnoses included de novo or secondary AML, myelodysplastic syndrome, and myelofibrosis. All received peripheral blood stem cells from matched or mismatched unrelated donors, HLA-matched siblings, or haploidentical relatives. The FT14 protocol comprised fludarabine 150 mg/m^2^ over five days and treosulfan 42 g/m^2^ over three days, with rabbit antithymocyte globulin (5 mg/kg) for unrelated grafts. Primary endpoints were neutrophil and platelet engraftment, donor chimerism, incidence of acute and chronic graft-versus-host disease (GVHD), overall survival (OS), disease-free survival (DFS), relapse, and treatment-related mortality (TRM). Kaplan–Meier, Cox regression, and Fine and Gray models were applied. **Results:** Median age was 59 years; diagnoses included de novo AML (43%), secondary AML (16%), MDS (25%), and MF (16%). Neutrophil and platelet engraftment medians were 10 and 12 days, respectively. Full donor chimerism (≥99%) was achieved by day 31. Grade III conditioning-related toxicity occurred in 3.2% of cases. Five-year cumulative incidences of grade II–IV acute GVHD and moderate/severe chronic GVHD were 37.6% and 30.6%. Median follow-up was 16.3 months; relapse occurred in 25.3%. Five-year OS and DFS were 71% and 49% overall (75.8% and 59% in CR1), with TRM of 15.3%. Disease relapse and acute GVHD independently predicted inferior OS, and acute GVHD predicted TRM. **Conclusions:** The FluTreo FT14 regimen achieves rapid engraftment, universal high donor chimerism, low severe toxicity, and durable survival, supporting its use as a myeloablative, reduced-toxicity conditioning option in myeloid malignancies.

## 1. Introduction

Treosulfan-based conditioning regimens have emerged as a promising alternative for allogeneic hematopoietic cell transplantation (allo-HCT), particularly in patients with acute myeloid leukemia (AML) and other myeloid malignancies [1,2]. Traditionally, myeloablative conditioning (MAC) regimens relied on agents such as busulfan and cyclophosphamide, which, while effective, posed significant toxicity risks, including veno-occlusive disease and neurotoxicity [3]. However, many patients with myelodysplastic syndrome (MDS), especially older individuals or those with comorbidities, are not eligible for standard MAC due to unacceptable non-relapse mortality (NRM) rates. The introduction of reduced-intensity conditioning (RIC) regimens has expanded allo-HCT eligibility, allowing older patients to undergo transplantation without a marked impact of chronological age alone on outcomes [4].

Treosulfan, often combined with fludarabine, has demonstrated improved outcomes in terms of engraftment, overall survival, and reduced transplant-related mortality [5]. Compared to busulfan-based regimens, treosulfan offers a favorable toxicity profile, reducing complications while maintaining effective disease control. Additionally, studies suggest that treosulfan may provide superior cost-effectiveness while maintaining comparable or improved clinical benefits. A growing body of evidence supports its use in patients with MDS, where RIC regimens and advancements in supportive care have significantly reduced NRM and improved transplantation outcomes [6].

Beyond AML and MDS, treosulfan-based regimens are being explored in various patient populations, including those with primary immunodeficiency disorders. The increasing donor pool, facilitated by the wider use of alternative donors, has contributed to the marked rise in transplants for MDS over the past two decades, making MDS the third most common indication for allo-HCT in Europe. This trend underscores the importance of refining conditioning regimens to optimize patient outcomes while minimizing toxicity [7].

As research continues to evolve, ongoing clinical trials and retrospective analyses will further define the optimal use of treosulfan-based conditioning regimens. The increasing adoption of treosulfan highlights the need for continued investigation into long-term outcomes, patient selection criteria, and potential refinements in dosing strategies. With its promising profile, treosulfan is poised to become a cornerstone in conditioning regimens for allo-HCT, improving patient outcomes and expanding treatment accessibility.

## 2. Results

### 2.1. Patient Characteristics

Between January 2015 and December 2023, 186 consecutive patients received FT14 conditioning and allo-HCT. The median age was 59 years (range, 18–70; mean, 57.3 ± 8.53). Underlying diagnoses were de novo AML (n = 80; 43.0%), secondary AML (n = 30; 16.1%), MDS (n = 46; 24.7%), and (MF) (n = 30; 16.1%). At transplant, 89 patients (47.8%) were in first complete remission (CR1) and 62 (33.3%) in CR2, and 35 (18.8%) had refractory or relapsed disease. Donor sources comprised matched unrelated (n = 90; 48.4%), mismatched unrelated (n = 23; 12.4%), HLA-matched sibling (n = 59; 31.7%), and haploidentical family donors (n = 12; 6.5%). A summary of patient characteristics at transplantation are shown in Table 1.

### 2.2. Engraftment, Chimerism, and Early Toxicity

All patients achieved neutrophil engraftment at a median of 10 days (range, 8–13; mean, 10.11 ± 1.13) and platelet engraftment at a median of 12 days (range, 9–55). Full donor chimerism (≥99%) was documented in every patient at a median of 31 days post-transplant (range, 14–92). Median length of stay in the BMT unit was 18 days (range, 10–29; mean, 17.62 ± 3.69). Grade III diarrhea occurred in six patients (3.2%); no other grade III–IV conditioning-related toxicities were observed.

### 2.3. Graft-Versus-Host Disease

The 5-year cumulative incidence (CI) of grade II–IV acute GVHD was 37.6% and the CI of moderate/severe chronic GVHD was 30.6%. In multivariate analysis, the occurrence of grade II–IV acute GVHD was independently associated with inferior overall survival (*p* = 0.001) and was the sole independent predictor of treatment-related mortality (hazard ratio, 0.27; 95% CI, 0.11–0.64; *p* = 0.003). No other evaluated variables, including disease type, donor source, the number of prior treatment lines, and disease phase at transplantation, demonstrated a statistically significant association with TRM. These findings underscore the clinical impact of acute GVHD on long-term outcomes in this cohort.

### 2.4. Relapse, Survival, and Treatment-Related Mortality

**Figure 1:**
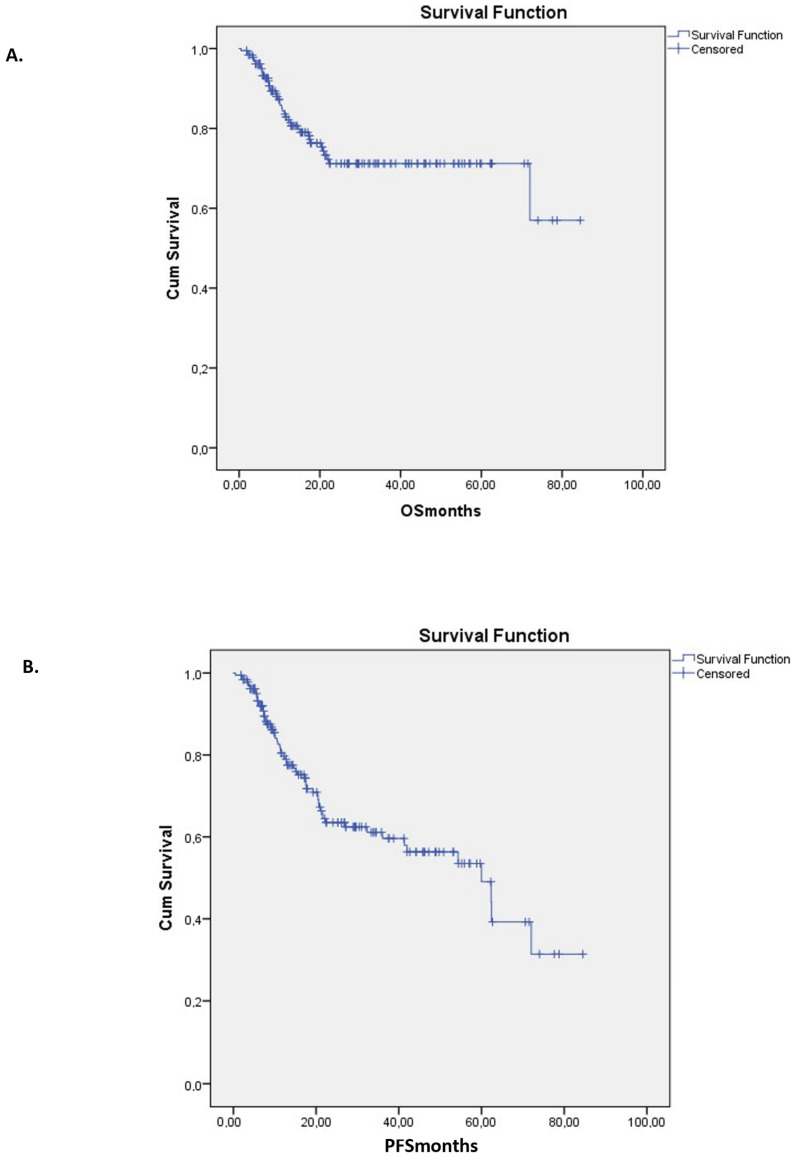
Kaplan–Meier curves for overall (**A**) and disease-free (**Β**) survival. In this Graph, Cumulative as “Cum”, moths of follow-up as “OSmonths” and “PFSmonths” respectively.

**Figure 2:**
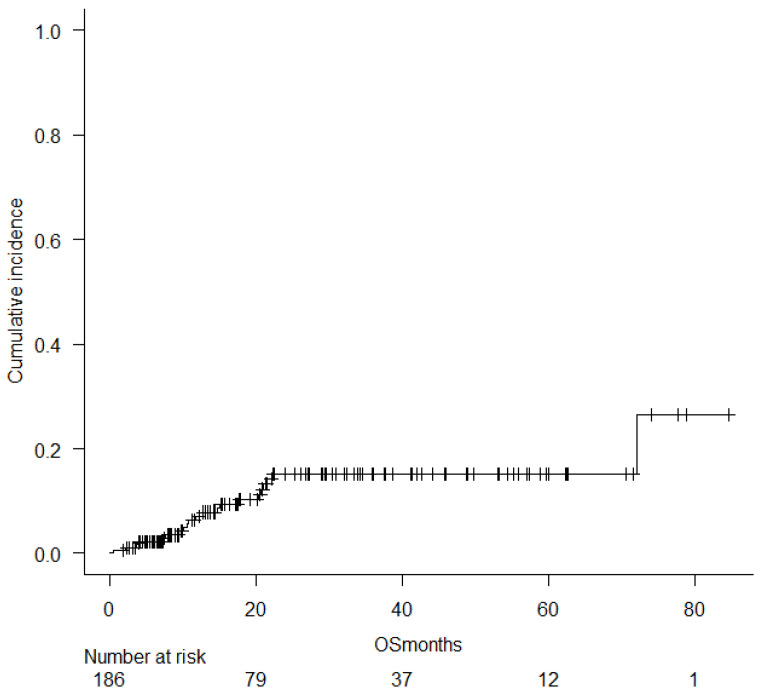
Five-year cumulative incidence of treatment relapse mortality. In this Graph, moths of follow-up as “OSmonths”.

With a median follow-up of 16.3 months (range, 1.5–84.5; mean, 22.8 ± 19.5), 47 patients (25.3%) relapsed. Five-year OS for the entire cohort was 71% (Figure 1A), and 5-year DFS was 49% (Figure 1B). Among patients transplanted in CR1, 5-year OS was 75.8% and DFS was 59%. The 5-year CI of TRM was 15.3% (Figure 2).

Five-year OS for the entire cohort was 71% (Figure 1A; 76%, 61%, and 64% (*p* = NS) for AML, MDS, and MF, respectively), and 5-year DFS was 49% (43%, 51%, and 52% (*p* = NS) for AML, MDS, and MF, respectively).

The 5-year CI of TRM was 15.3%, with TRM at day 100 being present in 5.1% of cases (16.2%, 13.2%, and 17.2% (*p* = NS) for AML, MDS, and MF, respectively), and causes of death included acute GVHD (75%), severe infections (19%), and graft failure (6%).

### 2.5. Multivariate Analyses

In a Cox regression model for OS, both disease relapse (*p* < 0.001) and grade II–IV acute GVHD (*p* = 0.001) were independent predictors of inferior survival; disease phase at transplant did not significantly affect OS (*p* = 0.144) (Table 2).

Fine and Gray competing-risk regression identified grade II–IV acute GVHD as the sole independent predictor of TRM (hazard ratio, 0.27; 95% CI, 0.11–0.64; *p* = 0.003). Other factors, including disease type, donor source, the number of prior treatment lines, and disease phase, were not significantly associated with TRM (Table 3).

## 3. Discussion

Treosulfan-based conditioning regimens have emerged as a promising alternative to conventional myeloablative and busulfan- or TBI-based conditioning for allo-HCT in AML, MDS, CMML, MF, and other myeloid malignancies. Across several studies, treosulfan, especially when combined with fludarabine (FluTreo regimen), demonstrates substantial antileukemic activity while reducing TRM and NRM.

Our real-world study of 186 patients under the conditioning regiment FT14 (fludarabine 150 mg/m^2^–treosulfan 42 mg/m^2^), with a mean age of 57.3 years (95% CI, 56.07–58.54) and a balanced combination of AML, MDS, and myelofibrosis, predominantly transplanted in first or second complete remission, validates rapid engraftment with neutrophils at a mean of 10 post-transplant days (95% CI, 9.78–10.44) and platelets at a median of 12 post-transplant days (range, 9–55), as well as universal attainment of full donor chimerism within a median of 31 post-transplant days (range, 14–92). The regimen’s safety is underlined by incredibly low rates of severe toxicity (only 3.2% presented grade 3 diarrhea) alongside acceptable rates of acute (37.6%) and moderate/severe chronic (30.6%) GVHD, a 60-month TRM incidence of 15.3% without statistically significant variation between multivariate analyses, and promising long-term outcomes with an overall 60-month OS of 71% (and 75.8% in CR1) and an overall 60-month DFS of 49% (and 59% in CR1). These outcomes complement the progressing evidence that treosulfan-based conditioning can offer robust disease control with reduced toxicity burden. While the heterogeneity of our cohort may limit disease-specific conclusions, it reflects the real-world patient population undergoing allo-HCT with FT14 conditioning. The consistent engraftment kinetics, low early TRM, and favorable survival outcomes across disease categories and donor types suggest that the regimen’s reduced-toxicity profile is broadly applicable.

Several studies have testified comparable or even superior findings using treosulfan-based regimens. Berning P et al., 2024, assessed FluTreo over 8Gy TBI/Flu in patients with AML and MDS and obtained similar survival [36-month OS of 71% (95% CI, 65–79)], relapse [21% at 36 months (95% CI, 15–27)], aGVHD III/IV [9.2% at 6 months (95% CI, 5.8–14%)], and cGVHD [41% at 36 months (95% CI, 34–48)] incidences [8]. Our results endorse these findings, as our OS and DFS outcomes line up with those achieved by TBI-based regimens, underscoring that the FT14 regimen demonstrates substantial antileukemic activity while diminishing regimen-related toxicity. In addition, Deeg HJ et al., 2018, investigated modifications to treosulfan-based regiments, such as the addition of low-dose TBI to further reduce relapse, particularly in AML patients (14% vs. 35%) [9]. While our study did observe a 25.3% relapse rate overall, this was within the range conveyed by similar studies [21% at 36 months (95% CI, 15–27) by Berning P et al., 2024 [8]; 20% at 12 months (95% CI, 14–25) by Gagelmann N et al., 2024 [10]; 20% at 36 months (95% CI, 11–29) by Robin M et al., 2024 [11]; 15.6% at 24 months (95% CI, 7.1–29.4) by Pasic I et al., 2024 [1]; 36% at 36 months (95% CI, 25–52) by Finke J et al., 2022 [12]; 25% at 60 months (95% CI, 21–30) by Shimoni A et al., 2021 [13]; 44.5% at 144 months (95% CI, 34.9–53.6) by Lazzari L et al., 2021 [14]; 24.6% at 24 months (95% CI, 17.8–31.3) by Beelen DW et al., 2020 [15]; 14.8% at 36 months (95% CI, 5.8–27.8) by Wedge E et al., 2020 [16] and emphasized the need for continued refinement of dosing strategies to optimize balance between disease control and toxicity.

Furthermore, Pasic I et al., 2024, accentuated how the advancing stratification of conditioning regimens’ intensity, with treosulfan-based approaches offering lower NRM [9.9% at 12 months (95% CI, 3.0–21.8)] while maintaining disease control, portrays treosulfan as a feasible alternative, especially for older or MAC-ineligible patients [1]. Our study population, which comprised patients transplanted in various remission states (from CR1 to relapsed/refractory settings), establishes that even for increased-risk patients, the FT14 regimen yields encouraging survival outcomes with manageable toxicity profiles. In addition, Braitsch K et al., 2022, evaluated reduced-toxicity regimens (FluTreo vs. FLAMSA-RIC) and noted comparable overall survival (30% at 60 months) despite differences in patient characteristics [17]. Our observed outcomes reinforce the conception that treosulfan-based strategies are effective and well-tolerated even among older, heavily pretreated patients, although we recorded high OS rates. Moreover, Finke J et al., 2022, applied the potential use of treosulfan-based conditioning to settings such as second HCT, finding increased complete remission rates regardless of substantial baseline risk, with our inclusion of patients with refractory/relapse disease further supporting effective deployment of treosulfan-based regimens in demanding clinical contexts [12]. Additionally, numerous studies have focused on expanding transplant eligibility and, specifically, Sakellari I et al., 2021, and Shimoni A et al., 2021, illustrated that FluTreo conditioning extends the accessibility of a myeloablative approach to elderly and more comorbid patients, with outcomes comparable to those of standard MAC regimens [13,18]. In our cohort, with a median age approaching 60 years and a significant fraction of patients likely having additional comorbidities, the favorable survival outcomes and low toxicity indices significantly reinforce this approach.

Earlier research by Nagler A et al., 2017, and Sakellari I et al., 2017, laid the groundwork by determining that treosulfan-based regimens in AML yield long-term outcomes, with 60-month OS of 38% (95% CI, 33–42) and 12-month OS of 76%, respectively, commensurate with TBI- or busulfan-based regimens while minimizing organ toxicity, such as VOD and pulmonary complications [19,20]. Further, Lazzari L et al., 2021, buttressed the advancement towards more refined, toxicity-sparing conditioning protocols, a trend that our data imitate through rapid engraftment and minimal regimen-related adverse events [14]. Likewise, the encouraging survival profile achieved with treosulfan-based conditioning is reflected by two studies by Beelen DW et al., 2024 and 2020, where they testified that the FluTreo conditioning regimen resulted in a significantly lower 24-month NRM of 11.4% (95% CI, 7.0–15.9) and TRM of 12.1% (95% CI, 8.1–17.7) and a 24-month OS of 71.3% (95% CI, 63.6–77.6) compared with higher-intensity regimens such as BuCy or FluMel [21]. These findings align with our outcomes and with the multivariate analyses where relapse and grade 2–4 aGVHD strongly predicted inferior OS, risk factors that treosulfan-based conditioning regimens have appeared to diminish. Furthermore, Wedge E et al., 2020, authenticated improved outcomes over time after the administration of FluTreo conditioning in MDS patients, with an enhanced 12-month OS of 84% (95% CI, 74.3–94.9) and 36-month OS of 71% (95% CI, 56.5–89.1), which reverberates with our promising long-term survival outcomes [16]. Gran C et al., 2020, further exhibited a superior 60-month NRM of 17% (95% CI, 13–21) in upfront-treated patients with treosulfan, a result supported by our low 60-month TRM incidence of 15.3% [22].

In the context of myelofibrosis, retrospective studies by Gagelmann N et al., 2024, and Robin M et al., 2024, signify the correlation of treosulfan-based conditioning, particularly in reduced-intensity protocols, with lower NRM [19% at 12 months (95% CI, 14–24) and 18% at 36 months (95% CI, 9–27), respectively] and improved PFS [48% at 48 months (95% CI, 40–55) and 62% at 36 months (95% CI, 50–73), respectively] compared with busulfan-based regimens [10,11]. Although our cohort consisted of a heterogenous mixture of diseases rather than MF alone, our TRM and rapid donor chimerism align with these observations and indicate that the favorable toxicity of treosulfan extends across different hematological malignancies.

Our study has several limitations. First, the absence of a prospective control group limits direct comparisons with other conditioning regimens. Second, the predominance of patients in CR1 may have contributed to favorable survival outcomes, introducing potential selection bias. Third, donor heterogeneity (matched, mismatched, haploidentical) could have influenced engraftment and GVHD rates. Additionally, complete cytogenetic/molecular data were not uniformly available for all patients over the 9-year accrual period, due to evolving diagnostic standards and referral patterns. Finally, the median follow-up of 16.3 months restricts the ability to fully assess late relapse, chronic GVHD evolution, and long-term TRM.

## 4. Materials and Methods

### 4.1. Study Design and Patient Population

We conducted a prospective cohort study of consecutive adult patients undergoing allo-HCT at our Joint Accreditation Committee—International Society for Cellular Therapy and European Group for Blood and Marrow Transplant—accredited unit between January 2015 and December 2023. Eligible patients met the following selection criteria: (1) age of 18–70 years at HCT; (2) diagnosis of de novo or secondary AML, MDS, or MF; (3) availability of a sibling or unrelated donor (8/8-allele-matched or 7/8-allele- or antigen-mismatched confirmed by molecular typing of HLA-A, -B, -C, and -DRB1); (4) peripheral blood stem cells as graft source; and (5) FT14 as the conditioning regimen. All consecutive eligible patients were enrolled at the time of transplantation, with pre-specified eligibility criteria, conditioning regimens, and supportive care protocols. Data collection was contemporaneous with clinical care, and outcomes were prospectively recorded in our institutional transplant database. No retrospective case selection or post hoc inclusion occurred.

Patient demographics, comprehensive clinical evaluations, and disease trajectories were meticulously documented. At the time of transplantation, disease status was classified as first complete remission (CR1), second complete remission (CR2), or refractory/relapsed. CR in patients with AML was defined as <5% bone marrow blasts, absence of circulating blasts, and hematologic recovery, irrespective of measurable residual disease (MRD) status. In addition, patients who achieved a morphologic leukemia-free state (MLFS) or CR with incomplete hematologic recovery (CRi) were also classified under the CR category. For MDS, CR was defined as <5% bone marrow blasts, absence of circulating blasts, hemoglobin ≥ 11 g/dL, an absolute neutrophil count (ANC) ≥ 1.0 × 10^9^/L, and a platelet count ≥ 100 × 10^9^/L. In MF, CR required normalization of bone marrow morphology with resolution of fibrosis, full hematologic recovery, absence of circulating blasts or immature myeloid cells, and absence of organomegaly or extramedullary disease. Patients who did not meet the above remission criteria were categorized as having refractory or relapsed disease at the time of transplantation.

All grafts comprised unmanipulated peripheral blood stem cells (PBSCs) sourced from HLA-matched siblings, matched or mismatched unrelated donors, or haploidentical family members. The institutional review board approved the study protocol, and each patient provided written informed consent in accordance with the Declaration of Helsinki.

### 4.2. Conditioning Regimen

The FluTreo myeloablative, reduced-toxicity conditioning regimen comprised intravenous fludarabine at a dose of 30 mg/m^2^/day administered over five consecutive days as a 20 min infusion (day −6 to day −2), combined with treosulfan at a daily dose of 14 g/m^2^ delivered as a one-hour infusion on each of three successive days (day −5 to day −3). Fludarabine was administered prior to treosulfan, with an interval of 40 min between the two infusions. In patients receiving grafts from unrelated donors, in vivo TCD Tcell depletion with low doses of rabbit antithymocyte globulin (ATG) was added at a total dose of 5–7.5 mg/kg. To prevent infusion-related reactions to ATG, methylprednisolone (80 mg every eight hours) was administered on ATG infusion days in both cohorts, followed by rapid tapering.

Post-transplant, prophylactic granulocyte colony-stimulating factor was routinely employed in both regimens. Supportive care included platelet transfusions when platelet counts fell below 20 × 10^9^/L and red blood cell transfusions for hemoglobin levels under 8 g/dL. All patients received broad antimicrobial prophylaxis against bacterial, fungal, and viral pathogens, with trimethoprim-sulfamethoxazole used to prevent Pneumocystis jirovecii infection. Molecular monitoring for cytomegalovirus and Epstein–Barr virus reactivation guided the initiation of preemptive therapy in accordance with international standards [23,24].

### 4.3. GVHD Prophylaxis

The evaluation and grading of acute graft-versus-host disease (GVHD) were conducted according to the Glucksberg criteria, while chronic GVHD was assessed using the Sullivan et al. classifications [25,26]. Recipients of both sibling and matched unrelated donor grafts received cyclosporine A (CSA) combined with mycophenolate mofetil through post-transplant for GVHD prophylaxis on day +45. CSA trough concentrations were maintained between 100 and 200 ng/mL until day +90 for sibling transplants and until day +150 for unrelated donor transplants. In the absence of chronic GVHD signs, the CSA dose was tapered by 5% weekly thereafter.

### 4.4. Chimerism

Donor–recipient chimerism was monitored in unfractionated bone marrow by short tandem repeat (STR) fragment analysis on days +14, +30, +60, and +90. Complete donor chimerism was defined as ≥99% donor-derived cells.

### 4.5. Statistical Analysis

Statistical analyses were performed using IBM SPSS Statistics for Windows, version 29.0. Continuous variables were reported as medians with corresponding ranges, whereas categorical variables were summarized as frequencies. The normality of continuous data was evaluated to determine the use of either Student’s *t*-test or the Mann–Whitney U test for group comparisons. Associations among categorical variables were examined by chi-square testing. Missing baseline covariate data were rare; when present, such cases were excluded from multivariable models involving that variable. Multivariable relationships were explored via logistic regression modeling. Survival probabilities were estimated by the Kaplan–Meier method and compared using the log-rank test. Cumulative incidence functions in the presence of competing events were calculated with EZR software version 1.68 (http://www.jichi.ac.jp/saitama-sct/SaitamaHP.files/statmed.html; accessed on 16 July 2025) [27] and assessed by Gray’s test, with subdistribution hazards analyzed through Fine and Gray regression. For TRM, competing-risk regression (Fine and Gray test) was used with relapse as the competing event. Variables were selected based on univariate analysis, clinical relevance, and the prior literature. A two-sided *p* value of less than 0.05 was considered to denote statistical significance.

## 5. Conclusions

Our real-world findings are in strong concordance with a growing body of evidence that positions treosulfan-based conditioning regimens as a promising and feasible alternative pending longer-term follow-up to conventional strategies. The consistent outcomes across these studies, including rapid and durable engraftment, lowered regimen-related toxicity, reduced non-relapse mortality, and competitive survival outcomes, reinforce further development and prospective evaluation of treosulfan-based protocols. As the transplant community moves toward more individualized treatment paradigms, the FT14 regimen represents an important step in delivering potent conditioning with a favorable safety profile, thereby expanding transplant eligibility and improving outcomes for patients across diverse hematologic malignancies.

## Figures and Tables

**Table 1:** Patient characteristics at transplantation.

Characteristic	Category	n (%)
Age at transplant	Median (range), years	59 (18–70)
Underlying disease	De novo AML	80 (43.0)
	Secondary AML	30 (16.1)
	Myelodysplastic syndrome (MDS)	46 (24.7)
	Myelofibrosis (MF)	30 (16.1)
Disease status at transplant	First CR (CR1)	89 (47.8)
	Second CR (CR2)	62 (33.3)
	Refractory/relapsed	35 (18.8)
Donor	Matched unrelated	90 (48.4)
	Mismatched unrelated	23 (12.4)
	HLA-matched sibling	59 (31.7)
	Haploidentical family	12 (6.5)

**Table 2:** Multivariate model of overall survival (Cox regression).

Variable	Exp (B)	*p* Value	Confidence Interval (CI)
Disease Relapse (Yes/No)	0.147	0.000	0.121–0.452
aGVHD	0.360	0.001	0.252–0.563
Disease Phase	1.155	0.144	0.921–1.457

**Table 3:** Multivariate model of TRM (Fine and Gray regression modeling).

Independent Predictor	Hazard Ratio	*p* Value	Confidence Interval (CI)
aGVHD	0.2694	0.0031	0.1898–0.4892
Disease	0.9902	0.7400	0.8901–1.8933
Donor	1.2400	0.5100	0.7831–2.1894
Lines	0.8351	0.4600	0.5678–1.9832
Phase	1.1440	0.6700	0.7832–3.2312

## Data Availability

The data presented in this study are available on request from the corresponding author. Τhe data are not publicly available due to privacy or ethical restrictions.
